# Visual and spatial modulation of tactile extinction: behavioural and electrophysiological evidence

**DOI:** 10.3389/fnhum.2012.00217

**Published:** 2012-07-25

**Authors:** Chiara F. Sambo, Giuseppe Vallar, Paola Fortis, Roberta Ronchi, Lucio Posteraro, Bettina Forster, Angelo Maravita

**Affiliations:** ^1^Department of Psychology, City University LondonLondon, UK; ^2^Department of Neuroscience, Physiology and Pharmacology, University College LondonLondon, UK; ^3^Department of Psychology, University of Milano-BicoccaMilan, Italy; ^4^Neuropsychological Laboratory, IRCCS Istituto Auxologico ItalianoMilan, Italy; ^5^Centre for Neurocognitive Rehabilitation, University of TrentoRovereto (TN), Italy; ^6^Rehabilitation UnitSuzzara (Mantova), Italy

**Keywords:** attention, ERPs, hand crossing, multisensory, space, tactile extinction

## Abstract

Crossing the hands over the midline reduces left tactile extinction to double simultaneous stimulation in right-brain-damaged patients, suggesting that spatial attentional biases toward the ipsilesional (right) side of space contribute to the patients' contralesional (left) deficit. We investigated (1) whether the position of the left hand, and its vision, affected processing speed of tactile stimuli, and (2) the electrophysiological underpinnings of the effect of hand position. (1) Four right-brain-damaged patients with spatial neglect and contralesional left tactile extinction or somatosensory deficits, and eight neurologically unimpaired participants, performed a speeded detection task on single taps delivered on their left index finger. In patients, placing the left hand in the right (heteronymous) hemi-space resulted in faster reaction times (RTs) to tactile stimuli, compared to placing that hand in the left (homonymous) hemi-space, particularly when the hand was visible. By contrast, in controls placing the left hand in the heteronymous hemi-space increased RTs. (2) Somatosensory event-related potentials (ERPs) were recorded from one patient and two controls in response to the stimulation of the left hand, placed in the two spatial positions. In the patient, the somatosensory P70, N140, and N250 components were enhanced when the left hand was placed in the heteronymous hemi-space, whereas in controls these components were not modulated by hand position. The novel findings are that in patients placing the left hand in the right, ipsilesional hemi-space yields a *temporal advantage* in processing tactile stimuli, and this effect may rely on a modulation of stimulus processing taking place as early as in the primary somatosensory cortex, as indexed by evoked potentials. Furthermore, vision enhances tactile processing specifically when the left hand is placed in the hemi-space toward which the patients' attentional biases are pathologically directed, namely rightwards.

## Introduction

Perception of sensory stimuli (e.g., tactile, visual) can be impaired following unilateral brain damage. Patients with unilateral hemispheric damage may fail to report stimuli contralateral to the side of the lesion (contralesional) due to primary sensory deficits (hemianaesthesia, hemianopia; Ropper and Samuels, 2009), or to higher-order disorders of spatial attention and representation such as unilateral spatial neglect (USN; Kooistra and Heilman, 1989; Vallar et al., 1991a,b). USN is a complex neuropsychological disorder, more frequent and severe after damage to the right cerebral hemisphere, whereby patients fail to report stimuli presented in the contralesional side of space, and to explore that portion of space (Bisiach and Vallar, 2000; Halligan et al., 2003; Heilman et al., 2003; Husain, 2008). The distinction between the primary sensory and the higher-order components underlying the defective perception of tactile and visual contralesional single stimuli may be made through electrophysiological methods (Vallar et al., 1991a,b; Angelelli et al., 1996), which show evidence of preserved primary sensory processing in these patients. The role of USN-related pathological mechanisms in bringing about deficits of somatosensory and visual perception of single stimuli delivered in the contralesional side of space and the body is also suggested by the clinical finding that somatosensory and visual half-field deficits are more frequent after right than after left hemispheric lesions (Sterzi et al., 1993). This hemispheric asymmetry cannot be readily accounted for in terms of primary sensory deficits, suggesting instead a higher-order impairment related to the right side of the lesion, and to deficits of spatial representation and attention (Vallar, 1998). The USN-related component of the somatosensory deficits of right-brain-damaged patients, which results in a defective report of somatosensory stimuli delivered to the left side of the body, has been termed “somatosensory hemineglect” (Vallar, 1998). Patients with unilateral hemispheric lesions may also fail to report the contralesional tactile or visual stimulus only when an ipsilesional stimulus is presented at the same time. This deficit (extinction to double simultaneous stimulation, see reviews in Bisiach and Vallar, 2000; Driver and Vuilleumier, 2001; Heilman et al., 2003) is, as USN, more closely associated with right rather than with left brain damage, but may occur independently of USN signs such as the defective exploration of peripersonal space (e.g., Vallar et al., 1994; Vossel et al., 2011). Extinction has been interpreted as a deficit of the orientation of spatial attention (with an ipsilesional bias): it manifests under conditions of bilateral stimulation, in which the ipsilesional and contralesional stimuli undergo an exaggerate competition for spatial attentional resources (Driver et al., 1997), with the ipsilesional stimulus exerting a disproportionate attraction of attention (Bisiach and Vallar, 2000; Driver and Vuilleumier, 2001; Heilman et al., 2003). Sensory extinction may occur both within and between sensory modalities (Brozzoli et al., 2006).

As for the tactile domain, a further indication of a spatial, rather than purely sensory, component of the somatosensory deficits of right-brain-damaged patients has been provided by the finding that irrigating the left external ear canal with cold water, or the right canal with warm water (caloric vestibular stimulation) temporarily ameliorates somatosensory deficits and extinction to double simultaneous stimulation in right-brain-damaged patients (Vallar et al., 1990, 1993; Bottini et al., 2005). The finding that caloric stimulation improves many aspects of the USN syndrome (Vallar et al., 1997) concurs with the abovementioned evidence to suggest that somatosensory deficits may have non-sensory components, related to the impairment of the spatial representations of corporeal space, contributing to the perceptual awareness of tactile stimuli (Gallace and Spence, 2007; Vallar, 2007).

Finally, a converging source of evidence comes from studies that have manipulated the reference frames in which stimuli are encoded, through the posture of the participants' hands (Moscovitch and Behrmann, 1994; Smania and Aglioti, 1995; Aglioti et al., 1999; Bartolomeo et al., 2004) or knees (Bartolomeo et al., 2004), with the aim of disentangling the relative contribution of the somatotopic and higher-order spatial reference frames in modulating the somatosensory deficit caused by unilateral brain damage. Brain-damaged patients with tactile extinction fail to report somatosensory stimuli delivered to the contralesional side of either wrist, when both sides of the wrist are simultaneously stimulated, regardless of whether the patients' hands are positioned palm up or palm down (Moscovitch and Behrmann, 1994): namely, irrespective of hand posture, patients extinguish the left-sided stimulus, with reference to the spatial, not to the sensory (somatotopic), coordinate frames. Similarly, the ability of right-brain-damaged patients to detect left-sided stimuli (both single and associated with a simultaneous right-sided touch) improves when their hands are crossed over the mid-sagittal plane of the body, so that the left hand is placed in the right-hand side of egocentric space (ipsilesional) and vice versa for the right hand (Smania and Aglioti, 1995; Aglioti et al., 1999; Moro et al., 2004). Such improvement appears to be reduced under high attentional load conditions, namely when patients are required to monitor several body sites (i.e., cheeks, hands, and knees) for tactile detection (Bartolomeo et al., 2004). Furthermore, when the right hand of right-brain-damaged patients with left tactile extinction is placed in the left side of space, detection performance worsens, although the size of the effect appears minor compared to that found for the left, contralesional hand placed in the right side of space, as discussed above (Aglioti et al., 1999). Altogether, these results are important as they suggest that higher-order, spatial impairments contribute to somatosensory deficits and tactile extinction of right-brain-damaged patients.

As in the abovementioned studies participants were blindfolded, the contribution of viewing the stimulated hand to these somatosensory disorders remains unexplored. Spatial frames of reference are dominated by vision (Shore et al., 2002; Eimer, 2004; Röder et al., 2004), which is the most accurate sensory modality for spatial perception in humans (Rock and Victor, 1964; Eimer, 2004). Furthermore, crossmodal links between vision and touch (Botvinick and Cohen, 1998; Tipper et al., 1998; Taylor-Clarke et al., 2002; Maravita et al., 2003; Fiorio and Haggard, 2005; Serino et al., 2007), and between vision and proprioception (van Beers et al., 1996, 1999; Botvinick and Cohen, 1998; Graziano, 1999; Lloyd et al., 2003; Maravita et al., 2003) have been extensively shown, including the critical role of vision in determining limb position (van Beers et al., 1996), in localizing tactile sensations (Botvinick and Cohen, 1998; Graziano, 1999), and in attentional selection (Sambo et al., 2009). Accordingly, the prediction can be made that non-informative vision of the stimulated hand may modulate spatial effects on tactile detection in right-brain-damaged patients with USN and tactile extinction or somatosensory deficits.

In this study, performed in right-brain-damaged patients with USN and tactile extinction or somatosensory deficits, we tested (1) whether the position of the left hand in space, and the vision of that hand, affected the processing speed of tactile stimuli, and (2) the electrophysiological underpinnings of the effect of hand position. We specifically tested our hypotheses in this kind of patients since previous studies show that only right-brain-damaged patients with tactile extinction or somatosensory deficits, but not right-brain-damaged patients without tactile extinction or left-brain-damaged patients, are more accurate in reporting stimuli delivered to the left hand when their hands are crossed over the midline compared to when their hands are uncrossed: critically, under these conditions, the improvement is found for stimuli delivered to the left hand, which is placed in the right (heteronymous) side of space (Smania and Aglioti, 1995; Aglioti et al., 1999). We hypothesized that in right-brain-damaged patients with these deficits, latencies to unilateral touches delivered to the left hand are shorter when that hand is placed in the right (“heteronymous”), ipsilesional side of space, compared to the left (“homonymous”), contralesional side of space, with reference to the mid-sagittal plane of the body, particularly when the hand is visible (Experiment 1). Furthermore, by recording somatosensory event-related potentials (ERPs) we addressed the question of which stages of somatosensory processing are modulated by the spatial position of the left hand. To this aim, in one right-brain-damaged patient with tactile extinction and in two age-matched neurologically unimpaired control participants, we compared ERPs elicited by tactile stimuli delivered to the left hand placed in the heteronymous or homonymous sides of space (Experiment 2).

## Experiment 1: simple reaction time

### Methods

#### Participants

**Figure 1:**
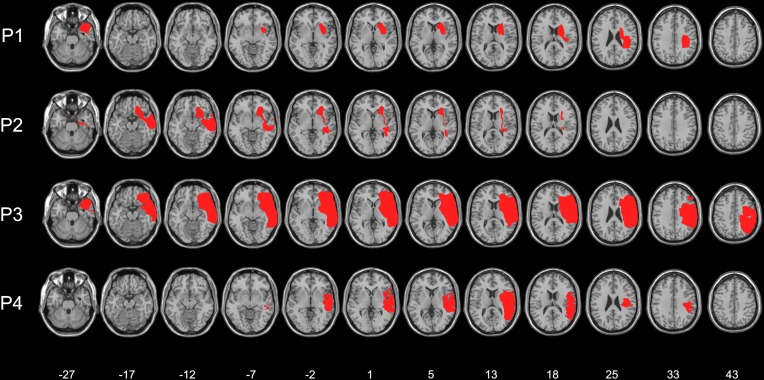
**Lesion maps of the four right-brain-damaged patients (see text for details).** Each individual lesion was superimposed onto a standard brain format conforming to stereotactic space. Montreal Neurological Institute (MNI) Z-coordinates of each transverse section are shown.

Four right-brain-damaged patients with left tactile extinction or somatosensory deficits (see details on the computerized somatosensory testing below) and USN (mean age: 62 years, see Tables 1 and 2), and eight age-matched, neurologically unimpaired control participants (mean age: 64.5 years, range: 31–87; mean years of education: 10.25, range: 3–17) entered in this study. Three patients were recruited from the Neuropsychological Laboratory of the IRCCS Istituto Auxologico Italiano, Milano, Italy, and one from the Rehabilitation Unit, Ospedale “C. Poma,” Bozzolo, Mantova, Italy. All patients, and the control participants, gave their informed consent to the study. All patients, and the control participants, were right-handed. Patients had no history or evidence of previous neurological or psychiatric disorders. The patients' demographic, neurological, and neuropsychological characteristics are summarized in Tables 1 and 2. Motor, somatosensory, and visual half-field deficit were assessed by a standard neurological exam (Bisiach and Faglioni, 1974). Figure 1 shows the lesion maps of the four right-brain-damaged patients who took part in Experiment 1. Patient #1, who also participated in Experiment 2, presented with a cortical-subcortical lesion affecting the basal ganglia (putamen and caudate nuclei), the temporal cortex, the rolandic operculum and, marginally, the parietal (post-central, supramarginal, and angular gyri) and inferior frontal cortices; the subcortical white matter was also extensively involved. Patient #2 had a surgical evacuation of an intracerebral hematoma and the lesion involved the frontal and temporal cortices, the basal ganglia, partially the insula and the white matter underneath the parietal and temporal cortices. Patient #3 had an extensive lesion, including the frontal (superior, middle, and inferior portions), parietal (post-central, angular, supramarginal, inferior, and superior regions), and temporal (superior, middle, and inferior portions) cortices, as well as the insula, the basal ganglia, and the subcortical white matter. The lesion of patient #4 involved the temporal cortex (superior, middle, and inferior portions), the frontal inferior regions, the parietal cortex (post-central, angular, supramarginal portions), the insula, the putamen, and the subcortical white matter.

**Table 1:** **Demographic and neurological characteristics of four right-brain-damaged, right-handed patients**.

**Patient**	**Sex/age**	**Schooling (years)**	**Aetiology/lesion site**	**Duration of disease (months)**	**Neurological deficits**		
					**M**	**SS**	**VF**
1	M/77	illiterate	I/BG/pvwm	14	1	2	2
2	M/36	9	#/H/BG/FT	12	1	0	0
3	M/76	17	I/FTP/pvwm	11	1	e	e
4	M/69	7	I/FTP	1	3	3	3

I/H, infarction, hemorrhage; #, surgical evacuation of an intracerebral hematoma; clamp of the middle cerebral artery. F, T, P, frontal, temporal, parietal cortico-subcortical damage; BG, basal ganglia; pvwm, periventricular white matter. Neurological impairment (M, motor; SS, somatosensory; VF, visual half-field): 1 (mild), 2 (moderate), 3 (severe) impairment, 0 (no deficit); e, extinction to double simultaneous stimulation.

**Table 2:** **Neuropsychological assessment scores**.

**Patient**	**Target cancellation**						**Line bisection (%)**	**Drawings**			**Personal neglect**		
	**Line**		**Letter**		**Bell**			**Complex**	**Daisy**	**Clock**		**L**	**R**
	**L**	**R**	**L**	**R**	**L**	**R**							
1	0/11	0/10	9/53^*^	2/51	2/18	0/17	+14,2^*^	9/10^*^	2/2	10/12	0/3^b^	13/15^f^	8/9^f^
2	7/11^*^	0/10	3/53	0/51	10/18^*^	4/17^*^	+11,2^*^	5/10^*^	1/2^*^	3/12^*^	0/3^b^	15/15^f^	9/9^f^
3	4/11^*^	0/10	53/53^*^	28/51^*^	18/18^*^	11/17^*^	+11,6^*^	5/10^*^	2/2	8/12^*^	0/3^b^	18/18^e^	n/a
4	n/a	n/a	42/53^*^	12/51^*^	18/18^*^	9/17^*^	+16,8^*^	7/10^*^	1.5/2	3/12^*^	0/3^b^	18/18^e^	n/a

Target cancellation: left-sided (L) and right-sided (R) omissions/number of targets; Line bisection, percent rightward deviation error; Drawings and Personal neglect: patient's score/maximum possible score (see text for details); n/a, not available or not applicable;

b , Bisiach's personal neglect test;

e , extension of Bisiach's personal neglect test;

f , Fluff test;

* , defective performance.

#### Neuropsychological assessment

USN was assessed using the following tests:

1. *Line cancellation* (Albert, 1973). The scores were the numbers of line targets crossed out by each participant (11 on the left-hand side and 10 on the right-hand side of the sheet). Marks such as lines, crosses, or dots systematically placed in the close proximity of each line were considered as correct cancellations. Neurologically unimpaired participants have a flawless performance on this task.
2. *Letter cancellation* (Diller et al., 1974). The patients' task was to cross out all of 104 H letters (53 in the left-hand side and 51 in the right-hand-side of the sheet), printed on an A3 sheet, together with other distractor letters. Neurologically unimpaired participants made a mean of 0.13 (0.12%, *SD* ± 0.45, range 0–4) omission errors out of 104 targets, with the maximum difference between omissions on the two sides of the sheet being two targets (Vallar et al., 1994).
3. *Bell cancellation* (Gauthier et al., 1989). The score was the number of “bell” targets crossed out by each participant (18 on the left-hand side and 17 on the right-hand side of the sheet). Neurologically unimpaired participants made a mean of 0.47 (1.3%, SD ± 0.83, range 0–4) omission errors out of 35 targets, with the maximum difference between omissions on the two sides of the sheet being four targets (Vallar et al., 1994).
4. *Line bisection*. The patients' task was to mark with a pencil the midpoint of six horizontal black lines (two 10 cm, two 15 cm, and two 25 cm in length, all 2 mm in width), presented in a random fixed order. Each line was printed in the center of an A4 sheet, aligned with the mid-sagittal plane of the participant's body. The length of the left-hand side of the line (i.e., from the left end of the line to the subject's mark) was measured to the nearest mm. That measurement was converted to a standardized score (percent deviation): measured left half minus objective half/objective half × 100 (Rode et al., 2006). This transformation yields positive numbers for marks placed to the right of the physical center, and negative numbers for marks placed to the left of it. The mean percent deviation score of 65 neurologically unimpaired participants, matched for age (mean 72.2, SD ± 5.16, range 65–83), and years of education (mean 9.5, SD ± 4.48, range 5–18), was 1.21% (SD ± 3.48, range –16.2 to +6.2%; Fortis et al., 2010).
5. *Five-element complex drawing* (Gainotti et al., 1972). The patients' task was to copy a complex five-element figure comprising, from left to right, two trees, a house, and two pine trees. Each element was scored 2 (flawless copy), 1.5 (partial omission of the left-hand side of an element), 1 (complete omission of the left-hand side of an element), 0.5 (complete omission of the left-hand side of an element, together with partial omission of the right-hand side of the same element), or 0 (no drawing, or no recognizable element). The horizontal ground line was not considered for scoring. The total score ranged from 0 to 10. The mean score of 148 neurologically unimpaired participants (mean age = 61.89, SD ± 11.95, range 40–89) was 9.89 (SD ± 0.23, range 9.5–10). Accordingly, a score lower than 9.5 indicated a defective performance (Mancini et al., 2011).
6. *Daisy drawing*. The patients' task was to copy a line drawing of a daisy. Scores ranged from 0 to 2 and were calculated as follows: 2 (flawless copy), 1.5 (partial omission of the left-hand side of the daisy), 1 (complete omission of the left-hand side of the daisy), 0.5 (complete omission of the left-hand side of the daisy, and partial omission of the right-hand side of the daisy), 0 (no drawing, or no recognizable element). The mean omission score of 148 neurologically unimpaired participants (mean age = 61.89, SD ± 11.95, range 40–89) was 1.99 (SD ± 0.12, range 1–2). Accordingly, the presence of a partial or complete omission of the left-hand side of the daisy (score lower than 1.5) was considered as indicative of left USN (Mancini et al., 2011).
7. *Clock drawing from memory*. The patients' task was to draw from memory the hours of a clock in a circular quadrant (diameter 12 cm), printed on an A4 sheet. Scores ranged from 0 to 12 and were calculated as follows: 1 (for each element in the correct position), 0 (for each omission or translocation of an element from one side to the other; elements “12” and “6” were scored as translocated when displaced in the right- or left-hand side quadrants). The mean score of 148 neurologically unimpaired participants (mean age = 61.89, SD ± 11.95, range 40–89) was 11.55 (SD ± 1.17, range 0–6). Accordingly, a score lower than 9 indicated a defective performance (Mancini et al., 2011). Furthermore, neurologically unimpaired participants made no translocations.
8. *Personal neglect* (Bisiach et al., 1986). The patients' task was to reach the contralesional hand with the ipsilesional hand (score range: 3 = maximum deficit, 0 = unimpaired performance). Two additional tests were also used: the Fluff test (Cocchini et al., 2001) in patients #1 and #2, and an extension of the personal neglect test (Bisiach et al., 1986; Fortis et al., 2010) in patients #3 and #4. In the Fluff test, the patients' task was to remove, with the right ipsilesional arm, 24 circle targets attached to the patients' clothes with velcro strap. The targets were located on the right-hand side (nine: three on the torso, three on the thigh, and three on leg) and on the left-hand side (15: three on the arm, three on the forearm, three on the torso, three on the thigh, and three on the leg) of the participants' body with respect to the midline. The number of collected items on both sides was scored (range 0–15 on the left, 0–9 on the right side of the body), for a total maximum score of 24. A score lower than 13 on the left-hand side of the body indicates defective performance (Cocchini et al., 2001). In the extension of the personal neglect test (Bisiach et al., 1986; Fortis et al., 2010), the patients' task was to reach six left-sided body parts (ear, shoulder, elbow, wrist, waist, knee), using their right hand. Each response was scored 0 (no movement), 1 (search without reaching), 2 (reaching with hesitation and search), or 3 (immediate reaching), with a 0–18 score range. Ten control participants made no errors (Fortis et al., 2010).

#### Tactile perception

The patients' ability to report single and double somatosensory stimuli was assessed by a computer-driven test (E-Prime, www.eprime2.eu). This consisted of 60 stimuli, with 20 tactile stimuli being delivered to the left hand, 20 to the right hand, and 20 bilaterally, in a random fixed order. Tactile stimuli were delivered using 12 V solenoids (www.heijo.com), driving a metal rod with a blunt conical tip that contacted the top segment of the index finger for 200 ms. Participants fixated a cross drawn on a paper sheet placed on the table where they rested their left arm; the fixation cross was aligned with the mid-sagittal plane of the participants' body, at a distance of about 50 cm. Participants received instructions to report verbally the occurrence and side of each delivered tactile stimulus (i.e., left-sided, right-sided, or bilateral). Patients were considered to show left-sided extinction when over 80% of unilateral left-sided tactile stimuli were reported correctly, and the left-sided stimulus of a bilateral stimulation was not reported in more than 30% of trials. The patients' performance is shown in Table 3. Three out of four patients showed left tactile extinction, while patient #4 missed 85% of the unilateral left-sided stimuli. Errors on bilateral trials always (100%) consisted of left-sided omissions. All control participants performed at ceiling with both unilateral and bilateral stimuli. It is noteworthy that the computerized procedure used here to assess extinction was more sensitive than the standard manual confrontation task. In particular, patient #2, who exhibited no deficit of tactile perception at the standard neurological examination, showed 100% extinction at the computerized test.

**Table 3:** **Percent correct responses (“right-sided”, “left-sided”, or “bilateral”) to computerized tactile stimuli**.

**Stimulation**	**Right-sided**	**Left-sided**	**Bilateral**
Patient 1	90%	95%	10%
Patient 2	100%	85%	0%
Patient 3	100%	85%	0%
Patient 4	85%	15%	0%
Control group (average)	100%	100%	100%

#### Experimental study

**Figure 2:**
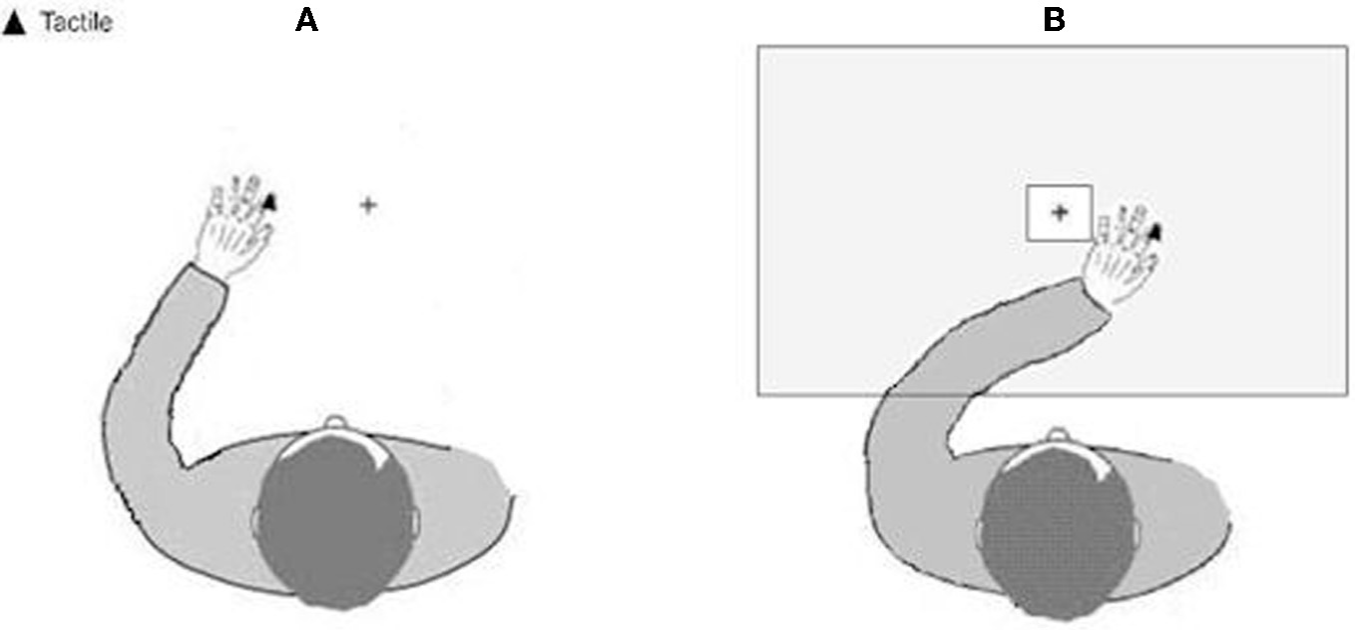
**Schematic representation of the experimental setup showing the position of the left hand. (A)** in the left-hand side of space (homonymous), and **(B)** in the right-hand side of space (heteronymous). Tactile stimuli were applied to the tip of the participants' left index finger.

A speeded tactile detection task was administered, consisting of eight experimental blocks, each including 40 trials. Tactile stimuli were delivered to the participants' left index finger in 30 trials per block. The remaining 10 were “catch trials” in which no stimulation was given. Tactile stimuli were delivered using a 12 V solenoid (see above), and consisted of single taps lasting for 200 ms. In alternating blocks, the participants' left hand was placed either in the left (“homonymous”) contralesional hemi-space, or in the right (“heteronymous”) ipsilesional hemi-space, with the vision of the left hand being either available or prevented. The right hand was always held along the body and hidden from view (see Figure 2). Participants performed four experimental conditions: “homonymous-seen”, “homonymous-unseen”, “heteronymous-seen”, and “heteronymous-unseen”. Two blocks were performed for each condition in an ABCDDCBA order (“homonymous-seen”, “homonymous-unseen”, “heteronymous-seen”, and “heteronymous-unseen”, then the reverse) for half of the participants, and the reversed order for the other half of the participants. A wooden box (70 × 35 × 10 cm) covered the participant's hands in the two “unseen” conditions. A central, squared aperture (side 15 cm) in the box allowed participants to see the fixation cross (see above). Participants were instructed to fixate the cross throughout each block, and make a vocal response (“one”) as quickly as possible whenever a tactile stimulus was detected. Vocal reaction times (RTs) were recorded by a voice key. Participants were allowed 2000 ms to respond after the stimulus presentation. Then the experimenter entered the participants' response (“1” when participants said “one,” and “0” for no response), and pressed a key on the computer keyboard for the next trial after checking for fixation, and ensuring that the participant was ready to proceed. Due to his low accuracy in the detection task, patient #4 completed two sessions of eight blocks each (i.e., 16 blocks in total), to provide enough trials for RTs analysis.

#### Statistical analysis

A repeated-measures ANOVA was performed in patients and control participants on the mean vocal RTs to tactile stimuli delivered to the left hand, with Hemi-space (two levels: “homonymous” vs. “heteronymous”) and Vision (two levels: “seen” vs. “unseen”) as within-subjects factors, and Group (two levels: “patients” vs. “controls”) as a between-subjects factor. Follow-up comparisons (*t*-tests and ANOVAs) were performed to explore significant two-way and three-way interactions.

### Results

**Figure 3:**
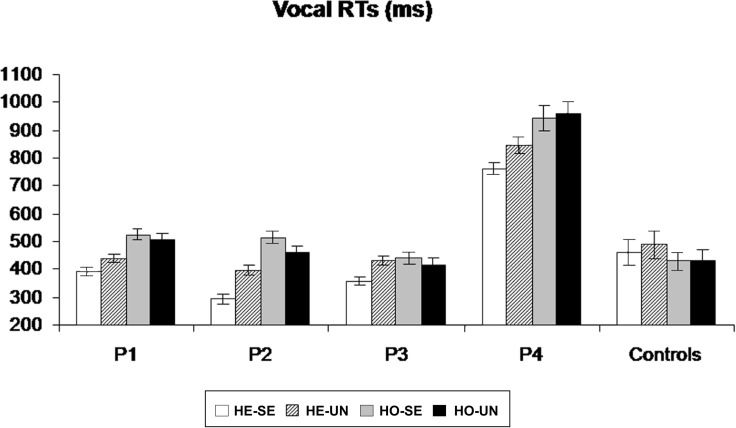
**Mean (and standard errors) vocal RTs to left-sided tactile stimuli for patients P1–P4, and for the control group, in the four experimental conditions, obtained by manipulating the hemi-space where the hand was placed (Homonymous/Heteronymous: HO/HE), and the vision of the left, stimulated hand (Seen/Unseen: SE/UN)**.

Patients #1, #2, and #3 and control participants missed on average less than 1% of tactile stimuli (range 0–2.2%). Patient #4 missed 44% of the stimuli in the “heteronymous-seen” condition, 46% in the “heteronymous-unseen” condition, 65% in the “homonymous-seen” condition, and 77% in the “homonymous-unseen” condition. The average false alarm rate for all participants (patients and controls) was 1.2% (range 0.3–2.4%). For each participant, trials in which the RTs exceeded ± 3 SD from the participant's average RTs were discarded. This procedure led to the removal of 2.3% of the trials overall. As shown in Figure 3, all patients were faster at responding to tactile stimuli in the “heteronymous” compared to the “homonymous” conditions. Moreover, all patients responded faster in the “heteronymous-seen” compared to “heteronymous-unseen” trials, while three out of four patients were slower in the “homonymous-seen” compared to the “homonymous-unseen” trials. On average, control participants were faster to respond to tactile stimuli under “homonymous” conditions, and showed a small advantage from seeing their left hand only in the “heteronymous” trials.

A repeated-measures ANOVA performed in patients and control participants on the mean vocal RTs to tactile stimuli delivered to the left hand revealed no main effect of Group [*F*_(1, 10)_ = 2.45, *p* = 0.24], indicating that, overall, patients' RTs were not significantly different from those of age-matched control participants. A main effect of Hemi-space was found [*F*_(1, 10)_ = 5.56, *p* = 0.043, η^2^= 0.46], with faster RTs to tactile stimuli on “heteronymous” (*M* = 481, *SD* = ± 168 ms) than on “homonymous” (*M* = 513 ms, *SD* = ±182 ms) trials overall. The main effect of Vision [*F*_(1, 10)_ = 8.17, *p* = 0.022, η^2^ = 0.57] was significant, indicating that participants were faster at responding to tactile stimuli when their hand was visible (*M* = 486, SD = ±187 vs. *M* = 509, SD = ±200 ms). The Group by Hemi-space interaction was significant [*F*_(1, 10)_ = 31.91, *p* = 0.001, η^2^ = 0.76], indicating that the response latencies in the patients were shorter for the “heteronymous” (*M* = 489, SD = ±202 ms) than for the “homonymous” (*M* = 585, SD = ±209 ms) trials, while control participants showed a reversed pattern (*M* = 474, SD = ±88 ms for “heteronymous” vs. 431, SD = ±76 ms for “homonymous” trials).

Follow-up ANOVAs were performed separately for the patients and the controls group, with the factors Hemi-space and Vision. These analyses revealed the presence of a significant main effect of Hemi-space on RTs in both groups [*F*_(1, 3)_ = 16.43, *p* = 0.013, η^2^ = 0.62 in the patients; and *F*_(1, 7)_ = 11.27, *p* = 0.017, η^2^ = 0.60 in the controls]. The opposite effects shown by the two groups (see above) confirm that control participants were significantly faster in the “homonymous” compared to the “heteronymous” trials, consistent with the literature (e.g., Yamamoto and Kitazawa, 2001), while the overall faster response in the “heteronymous” than “homonymous” trials found in the previous analysis was due to the large advantage of the patients in the former condition. A Hemi-space by Vision interaction was found in the patients' ANOVA [*F*_(1, 3)_ = 6.13, *p* = 0.04, η^2^ = 0.51], but not in the controls' ANOVA [*F*_(1, 7)_ = 2.33, *p* = 0.27]. Post-hoc t-tests in the patients, revealed significantly faster responses for the “heteronymous-seen” compared to the “heteronymous-unseen” trials [*t*_(3)_ = 4.78, *p* = 0.007], whereas the difference between “homonymous-seen” and “homonymous-unseen” trials was not significant [*t*_(3)_ = 1.78, *p* = 0.23]^1^.

## Experiment 2: somatosensory event-related potentials

### Methods

#### Participants

Somatosensory event-related brain potentials (ERPs) were recorded from patient #1 (see Table 1), and from two neurologically unimpaired age-matched male controls (Control #1, 78 year-old; Control #2, 80 year-old), who did not take part in Experiment 1. All participants gave written informed consent.

#### Experimental procedure

The general experimental set-up and procedures were similar to those of Experiment 1, with the following differences. First, vision of the left hand was available in all trials. Thus, participants performed the task under two experimental conditions, i.e., with the left hand placed in the left (“homonymous”) vs. the right (“heteronymous”) hemi-space (see Experiment 1), in alternating blocks. Second, in order to increase the number of critical left stimuli for the purpose of statistical analysis, a greater number of trials was given. Patient #1 was tested in two sessions, separated by 8 days. The two control participants completed one single experimental session. Each session consisted of eight blocks with 50 trials per block, including 40 left-sided touches and 10 “catch trials” (absent stimulation).

#### EEG recording and data analysis

**Figure 4:**
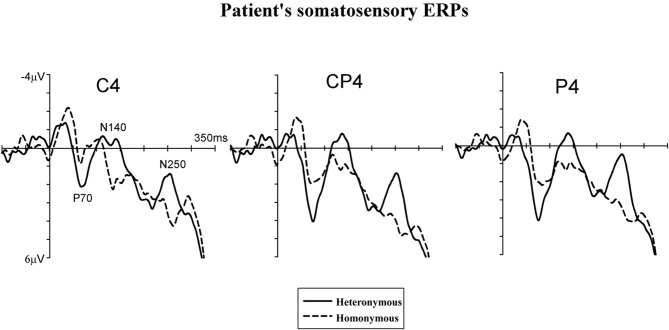
**Somatosensory ERP waveforms of patient #1.** Tactile stimuli were delivered to the left hand while this hand was placed in the right, heteronymous hemi-space (*solid lines*) and in the left, homonymous hemi-space (*dashed lines*). ERPs are shown in the 350-ms interval following stimulus onset for centro-parietal electrodes (C4, CP4, and P4) contralateral to the site of the tactile stimulation (i.e., over the right, damaged, hemisphere).

**Figure 5:**
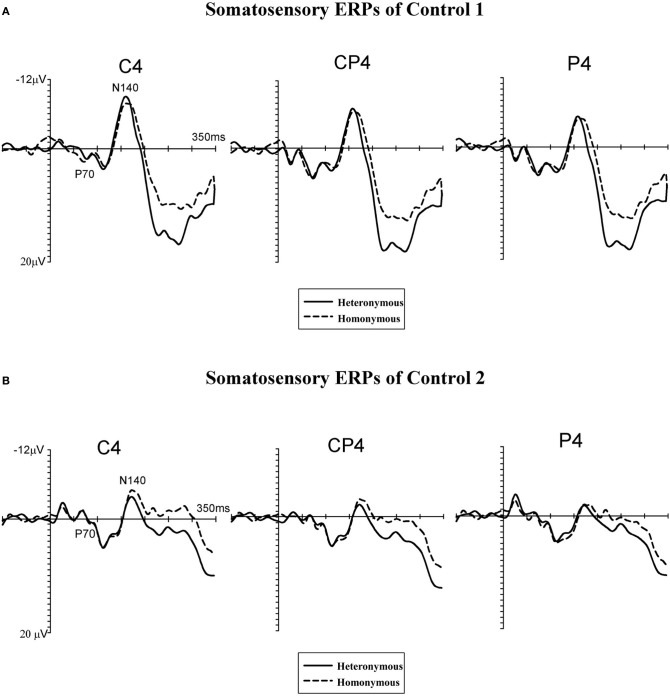
**Grand-averaged somatosensory ERP waveforms of two neurologically unimpaired participants (A,B).** Tactile stimuli were delivered to the left hand while this hand was placed in the right, heteronymous hemi-space (*solid lines*) and in the left, homonymous hemi-space (*dashed lines*). ERPs are shown in the 350-ms interval following stimulus onset for centro-parietal electrodes (C4, CP4, and P4) contralateral to the site of the tactile stimulation (i.e., over the right hemisphere).

EEG was recorded with Ag-AgCl electrodes from 28 scalp electrodes (midline electrodes: Cz, Pz, POz, Oz; electrodes over the right hemisphere: Fp2, F4, F8, C4, T8, TP8, Cp4, P4, P8, PO4, PO8, O2, and the homologous electrode sites over the left hemisphere). Horizontal electrooculogram (HEOG) was recorded bipolarly from the outer canthi of both eyes. Electrode impedance was kept below 5 kΩ. EEG and EOG were sampled with a 500 Hz digitization rate. EEG and EOG were epoched off-line into 450 ms periods, starting 100 ms before and ending 350 ms after the onset of tactile stimulation. Trials with eye blinks and movement-related artefacts (EEG waveforms exceeding ±80μV relative to baseline), measured at any recording sites within 350 ms after stimulus onset, were excluded from analysis. ERP waveforms were averaged relative to a 100 ms pre-stimulus baseline, separately for “homonymous” and “heteronymous” trials. The total number of trials contributing to the resulting average waveforms (collapsed across the two sessions) for patient #1 was 201 for “homonymous” and 189 for “heteronymous” trials. For statistical analysis each of the two sessions of the patient was further subdivided into two sub-sessions for a total of four sub-sessions for each experimental condition (“homonymous” vs. “heteronymous”). The mean number of trials contributing to the average ERPs for each sub-session was 62.75 (range: 54–78; for a similar statistical method see Marzi et al., 2000; Eimer et al., 2002). For the controls' data, each participant's session was subdivided into two sub-sessions, producing a total of four sub-sessions for each of the two left hand positions for the two participants. The mean amplitudes of early- and mid-latency somatosensory ERP components (P70^2^ and N140) were computed within analysis windows centered on the peak latency of these components. As the N140 component was somewhat delayed in both control participants compared to the N140 component observed in patient #1 (see Figures 4 and 5A,B), two distinct time windows were computed for this component centered on the peak of the N140 in the patient (N140p) and on the peak of the N140 in the controls (N140c). In addition, in order to investigate longer-latency effects of Hemi-space, the mean amplitudes were also computed within the analysis window centered on the peak latency of the patient's N250 component (N250p). This component was absent in the ERP waveforms of both control participants, who showed a “sustained negativity” beyond 220 ms post-stimulus. Thus, mean amplitude values were computed for the following post-stimulus latency windows in all participants: 55–90 ms post-stimulus (P70), 105–155 ms post-stimulus (N140p), 150–195 ms post-stimulus (N140c), 235–270 ms post-stimulus (N250p), and 220–350 ms post-stimulus. Analyses of ERP data were restricted to centro-parietal electrodes contralateral to the side of stimulation where somatosensory ERP components are maximal (Goff et al., 1978). Separate repeated-measures ANOVAs were conducted on mean amplitudes for the P70, N140p, N140c, and N250p components, and for the 220–350 ms post-stimulus measurement window with the factors Hemi-space (two levels: “homonymous” vs. “heteronymous”) and Electrode site (three levels: C4 vs. CP4 vs. P4) as within-subjects factors, and Group (two levels: patient's blocks vs. controls' blocks) as a between-subjects factor.

### Results

Figure 4 displays somatosensory ERPs recorded from patient #1 in response to left tactile stimuli delivered when the left (contralesional) hand was placed in the right, “heteronymous” (*solid line*) and the left, “homonymous” (*dashed line*) hemi-space. As can be seen from these waveforms, left tactile stimuli elicited a positive-going deflection peaking at about 70 ms after onset of the stimulus (i.e., somatosensory P70 component) followed by two negative deflections with a latency of about 140 ms (i.e., overlapping with the somatosensory N140 component), and 250 ms (i.e., overlapping with the somatosensory N250 component). As shown in Figure 4, tactile stimuli elicited enhanced P70, N140, and N250 amplitudes when the left hand was placed in the right hemi-space (“heteronymous” trials), compared to when the hand was held in the left hemi-space (“homonymous” trials). Similarly to the somatosensory ERPs recorded from one right-brain-damaged patient in a previous study (Eimer et al., 2002), somatosensory N80 and P100 components that are typically evoked by tactile stimuli in neurologically unimpaired participants (e.g., Michie et al., 1987; Taylor-Clarke et al., 2002; Eimer and Forster, 2003a) were not apparent in the patient's waveforms. Conversely, these components were present in the ERP waveforms of both control participants, following the P70 component (see Figures 5A,B). Importantly, Figure 5 suggests that in control participants none of the short- and mid-latency somatosensory components was modulated by the spatial position of the stimulated hand. In particular, the observation of the ERP responses suggests that in control #1 these components were not modulated by the hemi-space within which the hand was placed, while in control #2 the amplitude of the somatosensory N140 was, if anything, slightly larger for tactile stimuli delivered in the “homonymous” compared to “heteronymous” condition. This pattern is the reverse of that shown by the patient. In addition, at later time intervals a sustained negativity was evident in the waveforms of the control participants for tactile stimuli delivered when the left hand was placed in the “homonymous” compared to the “heteronymous” hemi-space, revealing a pattern opposite to that that shown in the patient's waveforms at a similar time interval.

Repeated-measures ANOVAs, performed on the somatosensory ERPs of the patient's and the controls' blocks, revealed a main effect of Group in the P70 [*F*_(1, 6)_ = 6.12, *p* = 0.041, η^2^= 0.47] and N140c [*F*_(1, 6)_ = 36.23, *p* = 0.001, η^2^ = 0.80] time windows, but not in the N140p [*F*_(1, 6)_ = 1.61, *p* = 0.23], N250 [*F*_(1, 6)_ = 1.97, *p* = 0.19], and 220–350 ms [*F*_(1, 6)_ = 1.49, *p* = 0.26] windows, indicating that the amplitude of the ERPs in the P70 and N140c time intervals was greater in the blocks recorded from control participants compared to those recorded from the patient. The main effects of Hemi-space and Electrode side were not significant in any of the time intervals tested (all *F*s < 1). The Group by Hemi-space interaction was significant in all time intervals tested except for the N140c interval [P70: *F*_(1, 6)_ = 7.15, *p* = 0.038, η^2^= 0.52; N140p: *F*_(1, 6)_ = 11.27, *p* = 0.019, η^2^ = 0.61; N140c: *F*_(1, 6)_ = 3.31, *p* = 0.32, η^2^ = 0.41; N250p: *F*_(1, 6)_ = 18.08, *p* = 0.006, η^2^ = 0.73; 220–350 ms interval: *F*_(1, 6)_ = 10.87, *p* = 0.021, η^2^ = 0.69]. The two-way interaction between Hemi-space and Electrode site and the three-way interaction between Group, Hemi-space, and Electrode site were not significant for any of the time windows tested (all *F*s < 1).

Follow-up ANOVAs were performed separately in the patient's and the controls' blocks for each of the time intervals to test the Group by Hemi-space interaction, with the factors Hemi-space and Electrode site. In the patient's blocks, a nearly significant effect of Hemi-space was found in the P70 [*F*_(1, 3)_ = 5.85, *p* = 0.052, η^2^ = 0.43]. The effect was significant in the N140p [*F*_(1, 3)_ = 6.70, p = 0.041, η^2^ = 0.50], and in the N250p [*F*_(1, 3)_ = 9.25, p = 0.024, η^2^ = 0.60] time windows, reflecting greater amplitudes of ERPs elicited by tactile stimuli in “heteronymous” compared to “homonymous” trials. In the latency range of the N140c component, and in the subsequent 220–350 ms post-stimulus interval, there was no main effect of Hemi-space [N140c: *F*_(1, 3)_ = 2.18, *p* = 0.16; 220–350 ms interval: *F*_(1, 3)_ = 2.98, *p* = 0.13]. There was a significant main effect of Electrode site in the P70 time window [*F*_(1, 3)_ = 6.29, p = 0.042, η^2^ = 0.53], but not in any of the other intervals tested (all *F*s < 1), indicating that the P70 component was overall smaller at the C4 electrode site compared to the other two electrode sites. The two-way interaction between Hemi-space and Electrode site was not significant for any of the time windows tested (all *F*s < 1). In the control participants, the same analyses did not show any main effect of Hemi-space for short- and mid-latency ERP components [P70: *F*_(1, 3)_ = 0.29, *p* = 0.43; N140p: *F*_(1, 3)_ = 0.78, *p* = 0.33; N140c: *F*_(1, 3)_ = 1.66, *p* = 0.23], indicating that no reliable differences in amplitudes were present at these latencies between ERPs elicited by tactile stimuli delivered when the left hand was placed in the “homonymous” vs. the “heteronymous” hemi-space. Similarly, in the latency range of the patient's N250 component (i.e., N250p) there was no main effect of Hemi-space [*F*_(1, 3)_ = 1.08, *p* = 0.28]. By contrast, a sustained negativity was elicited beyond 220 ms (i.e., 220–350 ms post-stimulus) by tactile stimuli in “homonymous” compared to “heteronymous” trials, resulting in a main effect of Hemi-space [*F*_(1, 3)_ = 6.10, *p* = 0.042, η^2^ = 0.52]. The main effect of Electrode site and the two-way interaction between Hemi-space and Electrode site were not significant for any of the time windows tested (all *F*s < 1).

## Discussion

All four right-brain-damaged patients were faster at responding to tactile stimuli delivered to their left hand when this hand was held in the right ipsilesional hemi-space. This finding confirms and extends previous observations showing that right-brain-damaged patients are more *accurate* in detecting left-sided tactile stimuli (under conditions of single and double stimulations) when their hands are crossed over the midline, so that the left hand is placed in the right (“heteronymous”) side of space, and vice-versa for the right hand (Smania and Aglioti, 1995; Aglioti et al., 1999; Moro et al., 2004). These results also add to previous evidence suggesting a crucial role for higher-order spatial and attentional factors, not only for sensory factors, in accounting for the somatosensory deficits exhibited by patients with tactile extinction and neglect (Vallar et al., 1990, 1997, 1993; Moscovitch and Behrmann, 1994; Vaishnavi et al., 2001; Gallace and Spence, 2007; Vallar, 2007). Processing of tactile stimuli by right-brain-damaged patients with extinction to double simultaneous stimulation may be slower for single unilateral stimulation, with increased latencies for stimuli presented in the left-hand side of space, compared to the right-hand side, under anatomical (uncrossed) hands posture (Eimer et al., 2002). A novel finding of the present study is that placing the left hand in the right-hand side of space yields a *temporal advantage* in the processing of tactile stimuli, compared to conditions in which that hand is held in the left-hand side of space. This pattern of results is in line with the view that conscious sensation of touch involves egocentric reference frames (Vallar, 1997, 1999), and tallies with a model proposed by Kitazawa (2002; based on data from neurologically unimpaired participants), which maintains that conscious sensation of touch is localized in space, namely at the location where the stimulated body part lies (in egocentric reference frames) before it is localized to the skin (in somatotopic reference frames; see also Azañón and Soto-Faraco, 2008).

Furthermore, we found that the temporal advantage given by placing the hand in the heteronymous side of space is significantly greater when patients are able to see their stimulated hand. In previous studies that manipulated hand position in order to investigate the role of somatosensory and spatial reference frames in tactile processing, right-brain-damaged patients (and so control participants) were blindfolded, as in a standard neurological examination of tactile sensation (Ropper and Samuels, 2009). Accordingly, both visuo-spatial information and vision of the hands were absent. Since in the present study visuo-spatial information was always available (that is, participants kept their eyes open throughout the experiment), our findings specifically suggest that seeing the left hand when placed in the right, ipsilesional side of space further facilitates processing of contralesional tactile stimuli in right-brain-damaged patients (see also Sambo et al., 2009). By contrast, vision of the left hand does not improve tactile detection when this hand lies in the left, “neglected” side of space. In fact, a perusal of the data from individual patients shows a decrease in performance (i.e., longer response latencies) in patients #1, #2, and #3 when vision is allowed and the left hand is held in the left hemi-space. Critically, while patient #1 presents with a left visual field defect, patient #2 has no left hemianopia, and patients #3 only shows visual extinction to double simultaneous stimulation. In right-brain-damaged-patients vision may further bias attentional resources toward the ipsilesional (right) side of space, reducing processing efficiency in the contralesional (left) side of space. The finding that USN symptoms may be more severe when vision is available, compared to conditions in which only tactile inputs are available (Gainotti, 2010; Mancini et al., 2011), is largely in line with these conclusions.

“Visual enhancement of touch,” that is, the facilitation of tactile processing by viewing the body, is observed specifically in difficult spatial discrimination tasks, but not in easier non-spatial task, in healthy participants (Press et al., 2004). Press and colleagues suggest that vision of the body improves tactile perception by enhancing the spatial representation of the body surface, which, in turn, may improve the signal-to-noise ratio for tactile processing. While in neurologically unimpaired participants this mechanism would be beneficial only under difficult task conditions, involving spatial discrimination (Press et al., 2004; Cardini et al., 2012), in right-brain-damaged patients with somatosensory deficits viewing the body may help tactile detection, possibly by recruiting a higher proportion of neurons, or increasing synchrony of neural firing, in response to the stimulation (McLeod et al., 1998). Such mechanisms are similar to those that have been proposed to be involved in spatial attention. Crucially, in our study the advantage shown by right-brain-damaged patients under viewing conditions occurs specifically when the left hand is placed in the right hemi-space, thus suggesting that viewing the body could further boost the advantage of placing the hand in the non-neglected (attended) hemi-space. Recently, two studies have specifically investigated the reciprocal effects of vision of a body location and attention to that location, in healthy volunteers. These studies have shown that these two effects may interact in such a way that visual information about the body facilitates spatial attentional selection of tactile input (Sambo et al., 2009; Michael et al., 2012) by enhancing activity within the somatosensory cortex. Here we provide the first evidence in patients with spatio-attentional deficits that vision enhances tactile processing specifically when the hand is placed in the hemi-space toward which attentional biases are directed (i.e., the right hemi-space, in right-brain-damaged patients with USN and tactile extinction or somatosensory deficits). We propose that, when the left hand is placed in the homonymous left hemi-space, contralateral to the patients' lesion, the representation of this side of space, which is mainly supported by the right (damaged, in right-brain-damaged patients) hemisphere (Bisiach and Vallar, 2000; Mesulam, 2002), fails to be, or is weakly, activated. Conversely, when the left hand is placed in the heteronymous right side of space, the representation of this side of space, mainly supported by the left (intact) hemisphere, may be activated, resulting in a higher processing speed of tactile stimuli applied to the left hand. Such space-based representations are controlled by fronto-parietal networks, that are also involved in multisensory integration between inputs from different modalities (e.g., touch, vision, and proprioception), and in the control of spatial attention (Mesulam, 2002; Maravita et al., 2003; Silver and Kastner, 2009; Vallar and Maravita, 2009).

In contrast with the pattern found in right-brain-damaged patients, control participants exhibit a disadvantage when their left hand is placed in the heteronymous hemi-space: their responses are significantly slower when the left hand is placed in the right, compared to the left, side of space. In a similar vein, previous studies in neurologically unimpaired participants show a reduction in perceived intensity and electrophysiological responses to somatosensory stimuli (Gallace et al., 2011), as well as a decrease in performance in temporal discrimination judgments (Yamamoto and Kitazawa, 2001; Shore et al., 2002), under crossed hands posture. In addition, in the present study the effect of vision of the stimulated hand on tactile detection is marginal and not significant in neurologically unimpaired participants, possibly because we did not use a difficult spatial tactile discrimination task (see Press et al., 2004).

In line with the behavioral results obtained in the patients' group, in one right-brain-damaged patient (#1) placing the left hand in the heteronymous side of space modulates somatosensory processing, as reflected by the enhancement of early- (i.e., P70) and mid-latency ERP (i.e., N140) components, as well as of a longer-latency component (i.e., N250), for left tactile stimuli delivered when the left hand is placed in the right hemi-space, compared to the left, “neglected,” side of space. According to intra-cranial recordings and MEG studies (Hari et al., 1984; Allison et al., 1992; Frot and Mauguière, 1999), somatosensory ERP components elicited within 100 ms, such as the P70, originate within SI, and the somatosensory N140 component originates in SII. The present results therefore suggest that holding the left hand in the “intact,” right-hand side of space may enhance neural activity in the primary somatosensory regions, which, in turn, facilitates detection of tactile stimuli delivered to that hand. In sum, spatial and attentional factors related to the position of the hand affect sensory cortical responses in patient #1. Previous studies in young neurologically unimpaired participants have also shown that spatial attention enhances the amplitude of short-latency somatosensory ERP and MEG components, starting as early as 40–50 ms after stimulus onset (Michie et al., 1987; Mima et al., 1998; Eimer and Forster, 2003a; Schubert et al., 2008). Residual activity has been observed in the SI and SII regions of the somatosensory cortex of the right hemisphere in patients with tactile extinction, during unilateral left, as well as bilateral, tactile stimulation (see Eimer et al., 2002 for an ERP study; and Remy et al., 1999 for a PET study). Such a residual processing may be boosted by placing the left hand in the “intact” right-hand side of space, allowing a more effective conscious elaboration of the sensory stimulus.

The present finding that the spatial position of the hand can modulate neural responses in early somatosensory areas is also in line with an fMRI study in a right-brain-damaged patient with mild left USN and left tactile extinction. In this study (Valenza et al., 2004), neural activity in the primary and secondary somatosensory areas was decreased when the patient's right ipsilesional hand was placed in the left (contralesional) side of space, as compared to when the hand was held in the right ipsilesional side of space (i.e., a manipulation opposite to the one used in the present study). Interestingly, fMRI responses were reduced under bilateral as well as unilateral tactile stimulation of the right hand in a crossed position (i.e., in the left-hand side of space). Behaviorally, however, the detection of touches to the right hand in a crossed position was dramatically reduced only when a simultaneous stimulation of the right elbow (placed in the right-hand side of space) was given. At the neural level, the results from Valenza et al.'s study (2004) suggest that the spatial position of body parts can modulate the strength of activation of early somatosensory areas also in response to single tactile stimulations, similarly to the results of the present study.

In addition to the modulation of early ERP components, enhancement of the patient's ERPs to tactile stimuli when the left hand was placed in the right, compared to the left, hemi-space is also present at later time intervals (i.e., around 250 ms after onset of the tactile stimuli, corresponding to the somatosensory N250 component). Such long-latency modulations are likely to stem from regions within the premotor frontal-posterior parietal network which are thought to be involved in the control of spatial attention (Mesulam, 1981; Corbetta et al., 1993; Gitelman et al., 1999; Hopfinger et al., 2000) and the spatial representation of the body (Schwoebel and Coslett, 2005; Tsakiris et al., 2007). In agreement with this view, greater activations of the posterior parietal cortex and of the middle frontal gyri were reported in the abovementioned fMRI study (Valenza et al., 2004) when the patient's right hand was held in the ipsilesional side of space (uncrossed position), compared to when it was placed in the left, contralesional side of space (crossed position). The increased processing of bodily stimuli through the integration of somatosensory, proprioceptive, and visual inputs from the stimulated body part (Rorden et al., 1999; Maravita et al., 2003; Vallar and Maravita, 2009) may also contribute to improve the patient's performance when the contralesional hand is crossed over the midline, so that the somatosensory input from that hand is made spatially coincident with the vision of the hand in the ipsilesional, intact visual field.

Unlike in patient #1, early somatosensory components in age-matched controls are not modulated by the spatial position of the left hand. However, a difference between ERPs in response to tactile stimuli emerged at later stages of processing, with a sustained negativity starting from about 220 ms after stimulus onset for stimuli delivered when the left hand was placed in the left, compared to the right, hemi-space, opposite to the pattern found in the patient. In previous ERP studies performed in healthy participants a sustained negativity was elicited at corresponding latencies by tactile stimuli presented at attended, compared to unattended, locations, indicating facilitation of processing for attended stimuli (Michie et al., 1987; Eimer and Forster, 2003a,b; Forster and Eimer, 2005). Our finding that, in neurologically unimpaired participants, tactile stimuli delivered to the left hand in the “homonymous” trials elicit an enhanced sustained negativity, compared to the “heteronymous” trials, may indicate increased attention allocated to the left hand when this is held in the left hemi-space (i.e., when the somatotopic and the spatial frames of reference overlap), compared to when that hand is placed in the right, heteronymous side of space. This is in line with the evidence that, in healthy participants, crossing the hands over the midline disrupts tactile-spatial selection processes, possibly because of the conflict between anatomical and external, visually defined spatial reference frames for coding body locations (Eimer et al., 2001; Heed and Röder, 2010).

It is important to note some limitations of this study. First, we investigated a limited number of patients. Therefore, although the present results provide insights into the effect of postural displacement and visual control of limbs on tactile processing in right-brain-damaged patients with USN and tactile extinction or somatosensory deficits, additional studies are needed to further qualify such effects and to understand the possible applications of these manipulations to clinical practice, for both the assessment and the treatment of tactile extinction and somatosensory deficits. Second, in this study we manipulated the spatial position and vision of the left hand but not of the right hand. Previous studies have shown that placing the right hand (Smania and Aglioti, 1995; Aglioti et al., 1999; Bartolomeo et al., 2004) or the right knee (Bartolomeo et al., 2004) in the left side of space slightly impairs tactile detection. However, such impairment is relatively small, and only occurs for double, but not single, stimulation conditions. Therefore, we may predict that, using our paradigm where only single tactile stimuli are delivered, especially in order to obtain clearer ERP data, no or minor effects would be found when manipulating the position of the right hand. Finally, in this study the performance of right-brain-damaged patients with tactile extinction was compared with that of age-matched unimpaired participants, but not with that of right-brain-damaged patients without tactile extinction or left-brain-damaged patients. Although it would be interesting to assess the performance of these participants, it is worth noting that Aglioti et al. (1999) showed that, unlike right-brain-damaged with somatosensory deficits and tactile extinction, right-brain-damaged patients without tactile extinction, as well as left-brain-damaged patients, are more accurate in reporting tactile stimuli when their hands are in the homonymous compared to the heteronymous position, that is, they perform similarly to neurologically unimpaired participants.

In sum, and keeping the abovementioned limitations in mind, the present behavioral and ERP results show that in right-brain-damaged patients with left USN and tactile extinction or somatosensory deficits, moving the left hand to the ipsilesional right-hand side of space improves somatosensory processing, possibly allocating more attentional resources to the tactile stimuli. The effects start from the very early stages of stimulus processing (putatively, in SI and SII), as indexed by an enhancement of early- and mid-latency somatosensory components (P70, N140) when the left hand is held in the heteronymous, compared to the homonymous, hemi-space. These findings may have clinical applications, not only for assessment but also for training to help recovery. Indeed, placing the left hand in the right, ipsilesional side of space may help differentiate primary somatosensory deficits from tactile extinction or USN in patients with right brain damage (e.g., Aglioti et al., 1999; Maravita, 2008). Secondly, the rehabilitation of somatosensory USN (Vallar, 1998) may be aided both by training the contralesional (left) hand while it lies in the right side of space, where the effect of any tactile stimulation may be enhanced, and by viewing the hand.

### Conflict of interest statement

The authors declare that the research was conducted in the absence of any commercial or financial relationships that could be construed as a potential conflict of interest.

## Footnotes

^1^Because of the more severe symptoms and the more acute stage of the illness of patient #4 compared to the other patients (see Tables 1 and 2), an additional ANOVA was conducted in the patients, with the factors Hemi-space and Vision, without including patient #4. A similar pattern of results was found in this analysis, with a main effect of Hemi-space [*F*(1, 2) = 18.64, *p* = 0.048] and a Hemi-space by Vision interaction [*F*(1, 2) = 19.35, *p* = 0.046].

^2^The P70 component may correspond to the P45 component observed in young neurologically unimpaired participants (e.g., Allison et al., 1992; Eimer and Forster, 2003a), here slightly delayed as in Eimer et al. (2002).
